# Supplementary material for: Skin Microbial Community Associated to Strawberry Disease in Farmed Rainbow Trout (Oncorhynchus mykiss Walbaum, 1792)
Source: Microorganisms. 2024 Jan 21;12(1):217. doi: 10.3390/microorganisms12010217 (PMC10818565; doi:10.3390/microorganisms12010217)
Supplement: Supplementary file 1 [file microorganisms-12-00217-s001.zip › microorganisms-2825371-supplementary.pdf]

Suplementary Material

Skin Microbial Community Associated to Strawberry Disease in Farmed Rainbow Trout (*Oncorhynchus mykiss* Walbaum, 1792)

Alda Pardo [1,2], Alejandro Villasante [1,3] and Jaime Romero [1,*]

Figure S1.- Macroscopic skin lesion caused by SD (Strawberry Disease).

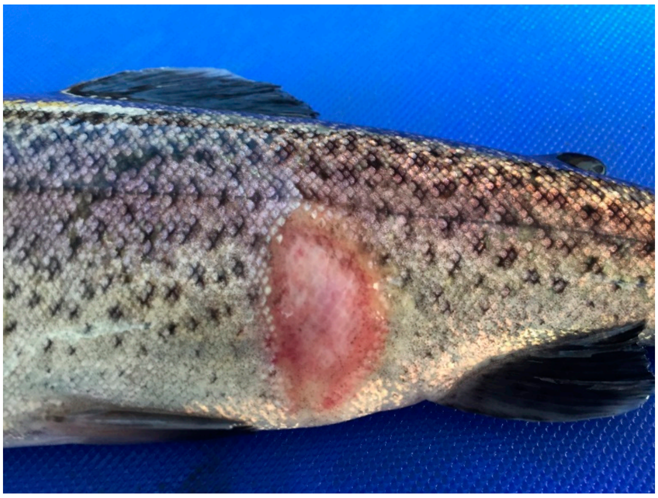

Figure S2.- Rarefaction curves of skin samples from trout with lesions (injury) and without lesions (non-injury).

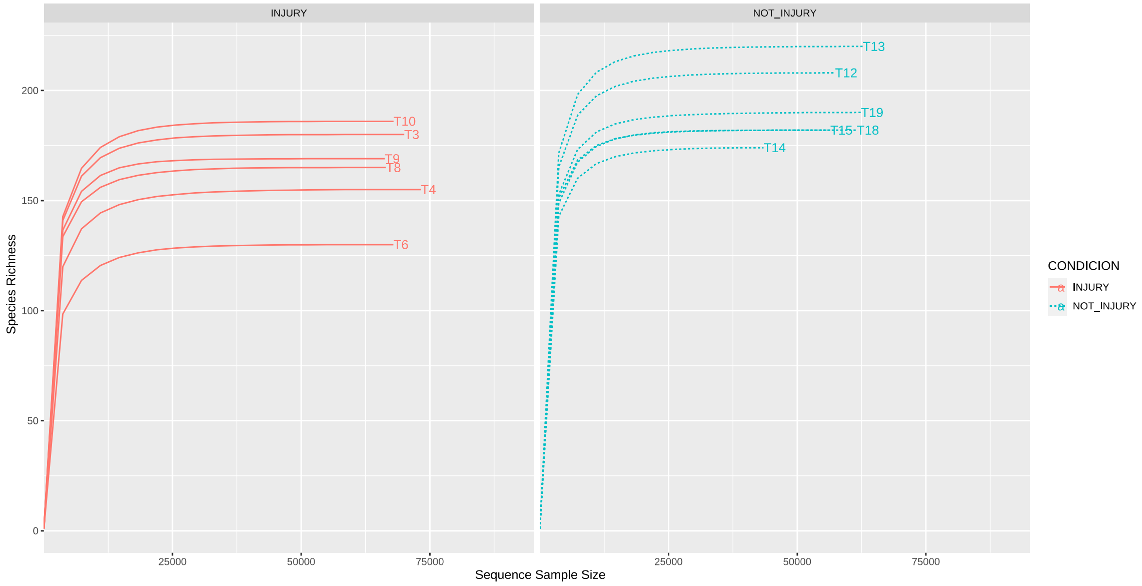

Table S1.- Highly similar sequences to the Candidatus Midichloria NGS sequence (ASV; 265 bp) were identified using megablast (blastn) in GenBank.

items	Accession Number	% identity	alignment length	mismatches	gap opens	Citation	PUBMED or DOI	Source/Organism
1	MN128406.2	100.000	265	0	0	Unpublished		environmental sample
2	EU555284.1	100.000	265	0	0	Lloyd 2008	19149374	skin rainbow trout
3	KT851867.1	99.245	265	2	0	Zaila 2017	28232825	Ichthyophthirius multifiliis
4	KT851863.1	99.245	265	2	0	Zaila 2017	28232825	Ichthyophthirius multifiliis
5	KT851862.1	99.245	265	2	0	Zaila 2017	28232825	Ichthyophthirius multifiliis
6	KT851804.1	99.245	265	2	0	Zaila 2017	28232825	Ichthyophthirius multifiliis
7	KT851759.1	99.245	265	2	0	Zaila 2017	28232825	Ichthyophthirius multifiliis
8	KT851803.1	98.872	266	2	1	Zaila 2017	28232825	Ichthyophthirius multifiliis
9	EU315771.1	95.849	265	11	0	Hornok 2008	18495345	flies and ticks
10	LC381237.1	95.472	265	12	0	Adenyo 2020	10.1016/j.actatropica.2020	ticks
11	MK416233.1	95.472	265	12	0	Selmi 2019	31398530	ticks
12	KY910125.1	95.472	265	12	0	Unpublished		ticks
13	MN783559.1	95.472	265	12	0	Unpublished		ticks
14	MN783558.1	95.472	265	12	0	Unpublished		ticks
15	LT575865.1	95.472	265	12	0	Unpublished		ticks
16	LT575864.1	95.472	265	12	0	Unpublished		ticks
17	LT575863.1	95.472	265	12	0	Unpublished		ticks
18	LT575860.1	95.472	265	12	0	Unpublished		ticks
19	OR295409.1	95.472	265	12	0	Unpublished		ticks
20	CP094379.1	95.472	265	12	0	Unpublished		ticks
21	OM982502.1	95.472	265	12	0	Sgroi 2022	35997363	ticks
22	OM982499.1	95.472	265	12	0	Sgroi 2022	35997363	ticks
23	HF568843.2	95.472	265	12	0	Unpublished		ticks
24	MZ476215.1	95.472	265	12	0	Unpublished		ticks
25	MZ476204.1	95.472	265	12	0	Unpublished		ticks
26	MZ476173.1	95.472	265	12	0	Unpublished		ticks
27	MZ476048.1	95.472	265	12	0	Unpublished		ticks
28	MZ475999.1	95.472	265	12	0	Unpublished		ticks
29	LR742713.1	95.472	265	12	0	Unpublished		ticks
30	LR742712.1	95.472	265	12	0	Unpublished		ticks
31	LR742711.1	95.472	265	12	0	Unpublished		ticks
32	LR742710.1	95.472	265	12	0	Unpublished		ticks
33	MW092747.1	95.472	265	12	0	Unpublished		ticks
34	MT965821.1	95.472	265	12	0	Unpublished		ticks
35	MT965820.1	95.472	265	12	0	Unpublished		ticks
36	FM992373.1	95.472	265	12	0	Beninati 2009	19732154	ticks
37	FM992372.1	95.472	265	12	0	Beninati 2009	19732154	ticks
38	AM411593.1	95.472	265	12	0	Epis 2008	18205982	ticks
39	EF687768.1	95.472	265	12	0	Venzal 2008	18071910	ticks
40	AF497583.1	95.472	265	12	0	Parola 2003	12682151	ticks
41	DQ379964.1	95.472	265	12	0	Loftis 2006	17004028	ticks
42	LC683106.1	95.094	265	13	0	Qiu 2022	35631087	ticks
43	LT898326.1	95.094	265	13	0	Unpublished		ticks
44	KX359181.1	95.094	265	13	0	Santos-Silva 2016	27894847	ticks
45	MN783572.1	95.094	265	13	0	Unpublished		ticks
46	MN783569.1	95.094	265	13	0	Unpublished		ticks
47	MN783566.1	95.094	265	13	0	Unpublished		ticks
48	MN783565.1	95.094	265	13	0	Unpublished		ticks
49	MN783564.1	95.094	265	13	0	Unpublished		ticks
50	MN783563.1	95.094	265	13	0	Unpublished		ticks
